# NCAM1 potentiates small cell lung cancer stemness and resistance to NK cell-mediated cytotoxicity

**DOI:** 10.1038/s41419-026-09182-2

**Published:** 2026-08-15

**Authors:** Jing Qi, Haiyan Sun, Yinli Yang, Jiaqi Zhang, Ran Zuo, Yenlie Chin, Zhansheng Jiang, Zhanyu Pan

**Affiliations:** 1 Department of Integrative Oncology, Key Laboratory of Cancer Prevention and Therapy, Key Laboratory of Cancer Immunology and Biotherapy, Tianjin’s Clinical Research Center for Cancer, Tianjin Medical University Cancer Institute and Hospital, National Clinical Research Center for Cancer, Tianjin, China; 2 Department of Radiotherapy, Key Laboratory of Cancer Prevention and Therapy, Key Laboratory of Cancer Immunology and Biotherapy, Tianjin’s Clinical Research Center for Cancer, Tianjin Medical University Cancer Institute and Hospital, National Clinical Research Center for Cancer, Tianjin, China

**Keywords:** Small-cell lung cancer, Tumour immunology

## Abstract

Intrinsic resistance of small cell lung cancer (SCLC) to immunotherapy underscores our limited understanding of how tumors can evade immune responses. Here we demonstrated that neural cell adhesion molecule 1 (NCAM1, also called CD56) facilitates disease progression, tumor stemness, and resistance to NK cell-mediated cytotoxicity in SCLC. Mechanistically, cis-binding of NCAM1 with its ligand L1CAM on the surface of identical tumor cell occupied considerable antigenic epitopes of L1CAM, and thereby prevented L1CAM from trans-binding with CD56^+^ NK cells to form immune-synapse structures, which may render the tumor cells more resistant to NK cell-mediated cytotoxicity in SCLC. Additionally, CHMP4B, a member of the ESCRT complex involved in plasma membrane repair, were essential for SCLC stemness and NK cell-mediated cytotoxicity, and NCAM1 can protect SCLC cells from immune attack and stem cell renewal by interacting with CHMP4B. SCLC specimens with concurrent high expression of NCAM1 and CHMP4B exhibited lower cytotoxic immune cell infiltration, were located farther from cytotoxic immune cells, and were associated with inferior clinical outcomes. These findings reveal potential mechanisms of NCAM1 in sustaining SCLC stemness and resisting cytolytic attack, and provide a potential target of CHMP4B against self-renewal and immunoresistance.

**Figure:**
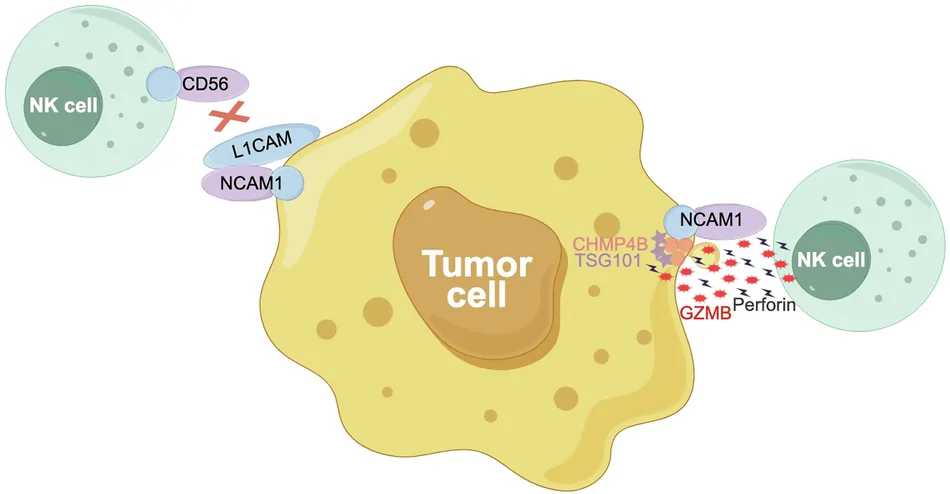

## Introduction

Although immunotherapy has brought survival benefits to patients with cancer, lung cancer remains the most malignant tumor in terms of highest mortality worldwide [1]. Small cell lung cancer (SCLC) is the most aggressive type of lung cancer, accounting for ~15–20% of all lung cancers [2]. Platinum-based chemotherapy combined with radiotherapy is the standard treatment for SCLC in clinical practice. Despite the encouraging initial treatment response, >95% of patients relapse or metastasize within 1 year, and the 5-year overall survival (OS) rate is below 7% [3]. Moreover, in fact, the addition of immunotherapy to standard chemotherapy only provides modest survival extension of ~15% of SCLC patients, and most patients cannot acquire long-term response [4]. Therefore, it is necessary to elucidate the intrinsic factors and their potential mechanisms causing resistance to immune cytotoxicity, which may provide optimized treatment strategies for clinical practice for the treatment of SCLC.

SCLC cells often upregulate the expression of multiple stemness-related genes, including CD133, SOX2, ABCG2, etc., whereby surviving and protecting tumor cells against chemoradiotherapy and eventually leading to disease progression [5, 6]. When it comes to immunotherapy, SCLC stem cells not only downregulate or lose the expression of MHC-I on their surface, enabling themselves unrecognizable by effector T lymphocytes and thus evading immune attack, but also upregulate the expression of immune checkpoint proteins such as PD-1 and LAG3 on the surface of T lymphocytes, inhibiting T-cell proliferation and activation [7, 8]. Meanwhile, cytokines released by cancer stem cells (such as TGF-β, GDF-15, IL-4, etc.) can directly inhibit the cytotoxic activity of cytotoxic T cells and/or recruit more immunosuppressive cells (such as MDSCs, Tregs, M2 macrophages, etc.) into tumor microenvironment, thereby sustaining immunosuppression continuously [9, 10]. Additionally, inflammatory cytokines such as IL-6 released by tumor-associated macrophages can even transform non-stem-like cells to stem-like cells by activating the STAT3 signaling pathway in SCLC cells [11].

NCAM1 (neural cell adhesion molecule 1), also known as CD56, belongs to the immunoglobulin superfamily of adhesion molecules and is one of the biomarkers for pathological diagnosis of SCLC in the clinic [12]. NCAM1 can trigger intracellular signaling cascades by interacting with components such as FGFR, N-Cadherin, and the extracellular matrix, playing an important role in neuronal differentiation and immune surveillance [13]. The widespread expression of NCAM1 was associated with poor outcomes in SCLC [13, 14]. However, due to the normal expression of NCAM1 in human tissues, such as neural cells and immune cells, antibody-drug conjugate targeting NCAM1 failed to improve patients’ survival given the severe adverse reactions, despite treatment response being observed in ~38% of SCLC patients with NCAM1 expression [13, 15, 16]. It has been reported that bone marrow mesenchymal stem cells isolated with NCAM1 monoclonal antibody show significantly enhanced colony-forming ability in vitro, indicating NCAM1 enhanced cell stemness [17]. Furthermore, NCAM1 is regarded as a biomarker to characterize cancer stem/progenitor cells, and elevated levels of peripheral soluble NCAM1 were associated with intrahepatic metastasis of hepatocellular carcinoma [18–20]. However, the role of NCAM1 in stemness maintenance and resistance to immune cytotoxicity has not been fully investigated in SCLC.

In the current study, we hypothesized that NCAM1 is required to support the self-renewal capacity of stem cells and may be an optimal molecule for investigating potential mechanisms of immune evasion. Our evidence demonstrated that the aberrant expression of NCAM1 on SCLC cells can result in suppression of target cell adhesion and lysis by NK cells, and NCAM1 can interact with endosomal sorting complex required for transport (ESCRT) members to more efficiently to greater efficiency in repairing perforin-induced membrane damage, thus enhancing tumor stemness and evading immune attack.

## Results

### NCAM1 is associated with SCLC progression and stemness

**Fig. 1:**
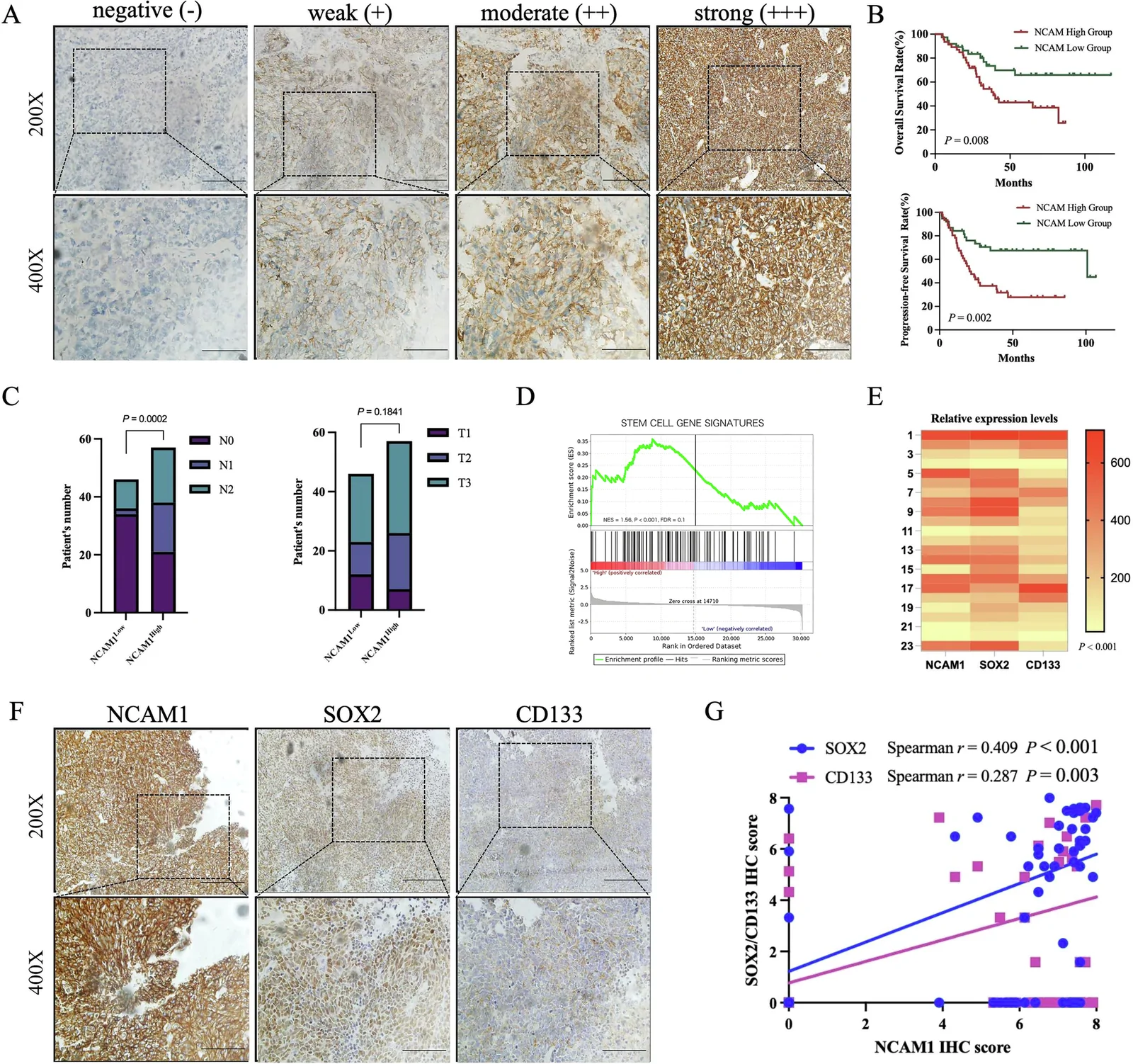
NCAM1 is associated with SCLC progression and stemness. **A** Representative IHC images of NCAM1 expression with distinct expression levels. Scale bar, 100 µm. **B** Kaplan–Meier analysis for OS (upper) and PFS (lower) of SCLC patients for different levels of NCAM1 using the Log-Rank test. **C** Distributions of T stage and N stage for SCLC patients with different NCAM1 expression. Chi-square test was used. **D** The GSEA enrichment of NCAM1^high^ and NCAM1^low^ group using the GDS4794 dataset. **E** Heatmap illustrating the relative expression abundance of NCAM1, SOX2, and CD133 using the GDS4794 dataset. Rows correspond to patient samples, while columns represent three stemness-related biomarkers. The color gradient bar on the right denotes the magnitude of relative expression values, ranging from low (pale yellow) to high (intense red). **F**, **G** Consecutive sections were stained with NCAM1, SOX2, and CD133 (**F**), and their expression correlation was displayed (**G**). Scale bar, 100 µm.

To investigate NCAM1 expression in primary SCLC tumors of humans, we performed immunohistochemical (IHC) staining using previously constructed tumor microarrays of 138 cases who underwent surgical resection when diagnosed (Supplementary Table 1) [21, 22]. According to the staining intensity and positive proportion, we dichotomized cases based on the median value of the staining result (intensity × proportion). Our analyses revealed that NCAM1 positively expressed in ~80% cases (Fig. 1A), and tumors with high NCAM1 expression exhibited inferior OS and progression-free survival (PFS) compared with low NCAM1 subgroup (Fig. 1B). Multivariable Cox regression analysis further confirmed the adverse effect of NCAM1 in SCLC prognosis after adjusting for stage, smoking history, and treatment (OS: hazard ratios, 1.005, 95% confidence intervals, 1.002–1.009, *P* = 004). In addition, patients with high NCAM1 expression harbored higher grade N stage, while not related to T stage (Fig. 1C). Gene enrichment analysis (GSEA) analyses showed significant enrichment of stem cell gene signatures in tumors with high level of NCAM1 (Fig. 1D), as exemplified by the positive correlations of messenger RNA (mRNA) levels among NCAM1, SOX2 and CD133 (Fig. 1E). Furthermore, the protein levels of NCAM1 were also positively correlated with SOX2 and CD133 expression (Fig. 1F, G), indicating the potential role of NCAM1 in labeling stem-like cancer cells in SCLC.

### NCAM1 promotes tumor stemness of SCLC

**Fig. 2:**
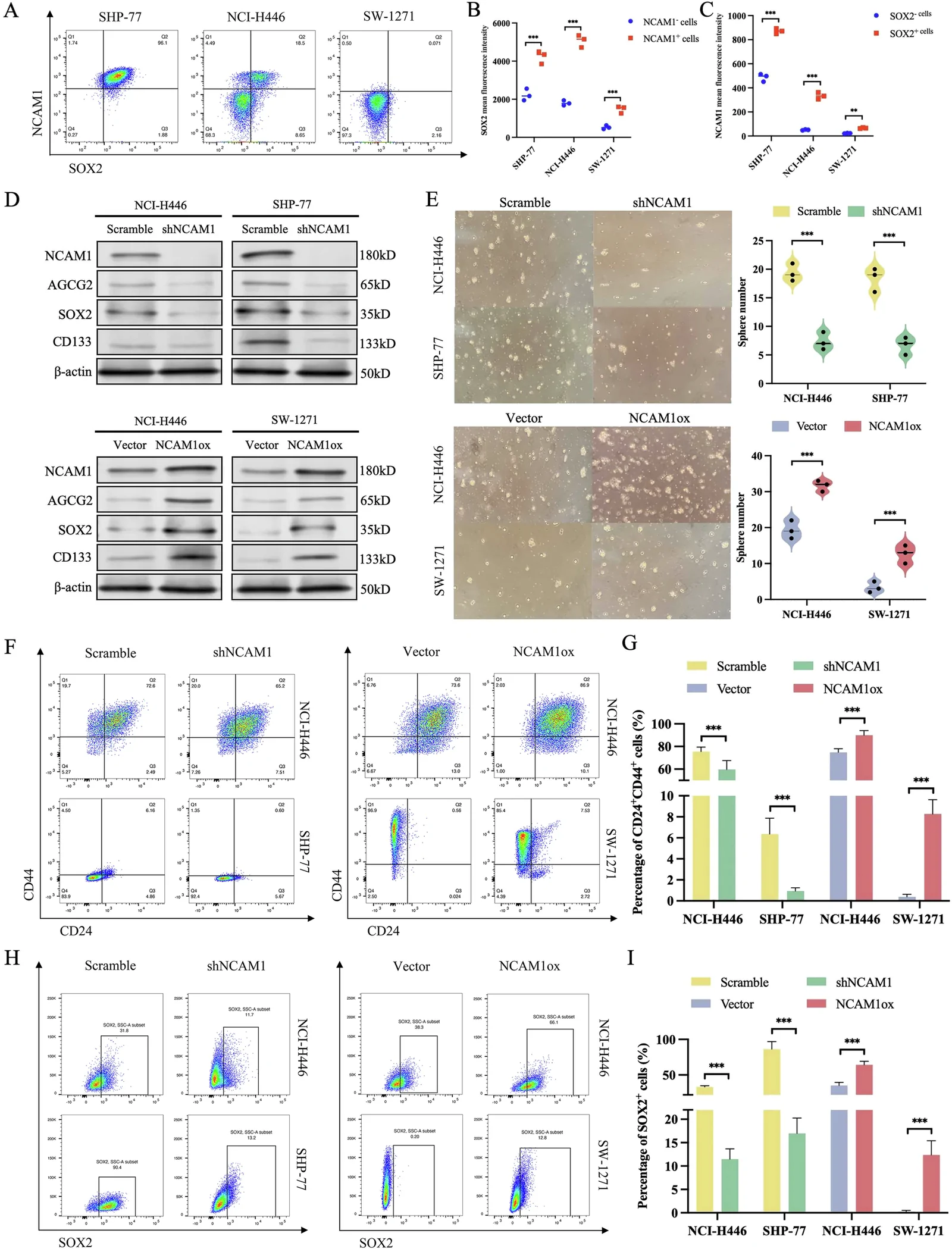
NCAM1 promotes tumor stemness of SCLC. **A** Indicated cell lines were stained with NCAM1 and SOX2, followed by flow cytometry detection. **B** Comparison of SOX2 MFI between NCAM1^+^ and NCAM1^−^ subsets in indicated cell lines. **C** Comparison of NCAM1 MFI between SOX2^+^ and SOX2^−^ subsets in indicated cell lines. **D** Western blottings for NCAM1, ABCG2, SOX2 and CD133 were analyzed in indicated cell lines under the loading control of β-actin. Representative results were displayed. **E** Sphere formation assays were performed in indicated cell lines. Representative images were shown (left) and sphere numbers were calculated (right). **F**, **G** Percentages of CD24^+^CD44^+^ cells in indicated cell lines were detected by flow cytometry (**F**) and the representative dot plots were shown (**G**). **H**, **I** Percentages of SOX2^+^ cells in indicated cell lines were detected by flow cytometry (**H**) and the representative dot plots were shown (**I**).

Considering the notable relationship between NCAM1 expression and stem-like phenotype of SCLC, we next examined the involvement of NCAM1 in regulating SCLC stemness in vitro. Herein, cancer stem cells were represented by the transcriptional factor SOX2. Three cell lines of SCLC were fluorescent labeled and subsequent analyses of flow cytometry found that the whole population in each cell line could be mainly divided into two subpopulations, namely NCAM1^+^SOX2^+^ and NCAM1^−^SOX2^−^ (Fig. 2A). Specifically, almost all SHP-77 cells were classified as the NCAM1^+^SOX2^+^ subset, while nearly all of SW_1271 cells belonged to the NCAM1^−^SOX2^−^ subset. These two subsets simultaneously appeared in NCI-H446 cells, with a substantial ratio of double-positive cells. Additionally, the mean fluorescence intensity (MFI) of SOX2 was markedly elevated in NCAM1^+^ relative to NCAM1^−^ subpopulations across all tested cell lines (Fig. 2B), and the MFI of NCAM1 was also significantly higher within SOX2^+^ compared to SOX2^−^ cell subsets (Fig. 2C), which revealed the strong association between the baseline NCAM1 expression and SCLC stemness.

To confirm the influence of NCAM1 on SCLC stemness, we transfected targeted lentiviral vectors into SCLC cell lines to stably overexpress (referred to as “NCAM1ox”) and silence (referred to as “shNCAM1”) NCAM1 levels, respectively. Cancer stem cell-associated markers, including ABCG2, SOX2, CD133, and CD24, and CD44, were used to evaluate the tumor stemness of SCLC. Western blotting experiment revealed substantial reductions in the expression of cancer stem cell-associated markers (ABCG2, SOX2, and CD133) after NCAM1 silencing (Fig. 2D). Consistently, silencing of tumor-supportive NCAM1 also decreased the proportions of CD24^+^CD44^+^ and SOX2^+^ stem cells of SCLC compared with those of the control group (Fig. 2F–I). On the contrary, the expression level of stemness-related proteins and the proportions of CD24^+^CD44^+^ as well as SOX2^+^ subsets were both increased in NCAM1 overexpression SCLC cells (Fig. 2D, G–I). Furthermore, in vitro tumorsphere cultivation assay demonstrated the stimulative capacity of NCAM1 in stem cell self-renewal (Fig. 2E). These results indicated that NCAM1 could promote tumor stemness of SCLC.

### NCAM1 suppresses NK cell-mediated cytotoxicity of SCLC

**Fig. 3:**
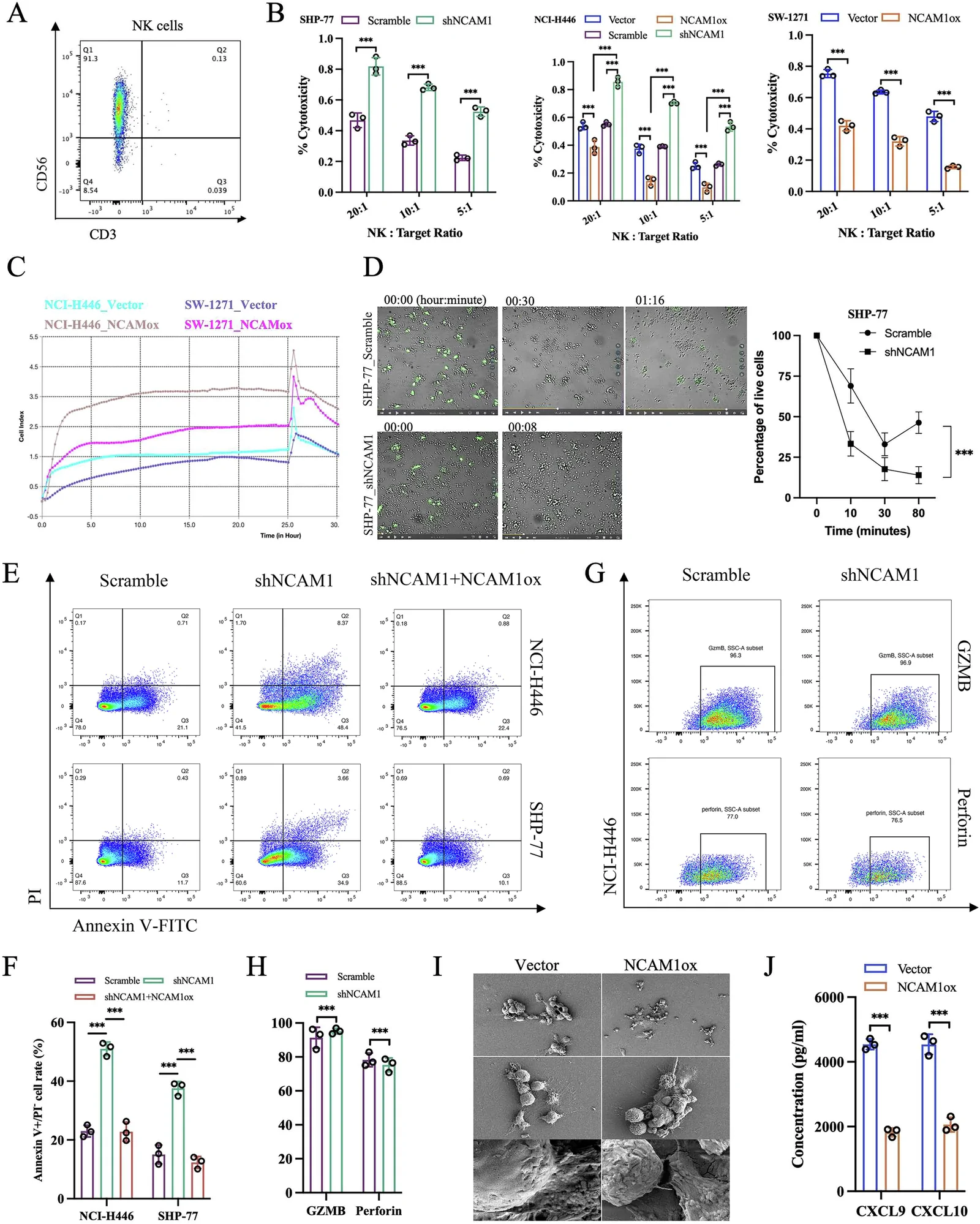
NCAM1 suppresses NK cell-mediated cytotoxicity of SCLC. **A** NK cells undergoing ex vivo activation and expansion were stained with CD3 and CD56, followed by flow cytometry to detect their positive rates. Representative dot plots were shown. **B** The lytic capacity of NK cells for the indicated cell lines was detected and analyzed at each ETRs. **C** A representative proliferation curve of the indicated cell lines was delineated with the co-incubation of NK cells. **D** NK cells were equally added to previously adhered cell lines, followed by dynamic live cell imaging. Represent imaging were displayed on the left, and summary data analysis was shown on the right. **E**, **F** NK cells were stained with NKG2D and then were equally co-incubated with indicated cell lines, followed by apoptosis cells labeling (annexin V and propidium iodide) and flow cytometry. NKG2D-negative cells were selected for analyzing apoptosis rates of indicated cell lines (**F**). Representative dot plots were shown (**E**). **G**, **H** NK cells were stained with NKG2D and were equally co-incubated with indicated cell lines thereafter, followed by flow cytometry to detect perforin and GZMB in NKG2D-positive cells (**H**). Representative dot plots were shown (**G**). **I** The immune-synapse structures formed by indicated tumor cells and NK cells were shown using a scanning electron microscope. Representative images were displayed. **J** Concentrations of CXCL9 and CXCL10 were detected and analyzed in the cell supernatant of co-incubation systems. Student’s *t* test was used.

Accumulated evidence suggested that cancer stem cells can evade immune recognization and response [23], we next sought to ask whether tumor-promoting effect of NCAM1 can resist immune cell-mediated cytotoxicity. Given that T lymphocyte cell-mediated immune reaction depended on the participation of type I major histocompatibility complex (MHC-I) and the downregulation of MHC-I was one of the mechanisms for most SCLC to escape immune response [7], we therefore selected natural killer (NK) cells to perform subsequent research, because MHC-I does not play a role in NK cell-mediated immune response. We magnetically separated, activated, and expanded NK cells from fresh peripheral blood mononuclear cells (PBMCs) of healthy individuals. After in vitro culture for 10-14 days, NK cells gradually expanded from the sorted single-cell state to form round-like cell clusters. Flow cytometry detected that the induction rate of mature NK cells (CD3^−^CD56^+^) was above 90% after in vitro expansion (Fig. 3A). Then, harvested NK cells were co-cultured with tumor cells at different effector-to-target ratios (ETR) in vitro. Cytotoxicity assays and realtime cellular analyses revealed that overexpression of NCAM1 impaired the tumor lysis capacity of NK cells and exhibited higher activity of tumor proliferation (Fig. 3B, C). By contrast, NCAM1-silenced cells were more sensitive to NK cell-mediated cytotoxicity at each ETR. In order to dynamically evaluate the lytic efficiency, we co-cultured NK cells with carboxyfluorescein succinimidyl ester (CFSE) pre-labeled tumor cells and performed live cell imaging. After silencing NCAM1, all of tumor cells were more rapid and efficient lysis by NK cells, while the control cells with intrinsic high expression of NCAM1 were inclined to resist NK cell-mediated lysis, and even a few tumor cells could evade the cytotoxic immune activity (Fig. 3D). Furthermore, we rescued the expression of NCAM1 in NCAM1-silenced cells, co-cultured with NK cells, and observed that NCAM1 rescue reduced NCAM1-induced tumor cell apoptosis in NCAM1-silenced cells (Fig. 3E, F). No significant difference in NK cell cytotoxicity (represented by GZMB and perforin) was observed after co-culturing with NCAM1-silenced and control tumor cells (Fig. 3G, H). These results demonstrated the adverse role of tumor-producing NCAM1 in NK cell-mediated cytotoxicity.

Considering that intercellular adhesion is a prerequisite for immune cells to exert their cytotoxic effects, we therefore administered scanning electron microscope (SEM) to examine whether tumorous NCAM1 can prevent the adhesion of NK cells with tumor cells. In the co-incubation system, we observed that most of the NCAM1-silenced cells were conjugated with NK cells, while a large proportion of NCAM1-overexpressed cells did not bind to NK cells at low magnification of SEM (Fig. 3I). Moreover, we also observed that NCAM1-silenced cells formed tight cellular junctions and immune-synapse structures with NK cells (Fig. 3I). Conversely, only a few NCAM1-overexpressed cells bound to NK cells (Fig. 3I). Notably, even the bound cells could not form tight cellular junctions or immune-synapse structures (Fig. 3I). Additionally, NK cells co-cultured with NCAM1-overexpressed cells released less chemokines of CXCL9 and CXCL10 (Fig. 3J), which may contribute to the role of NCAM1 in NK cytotoxicity resistance.

### Restriction of conjugate formation by cis-NCAM1/L1CAM interaction

**Fig. 4:**
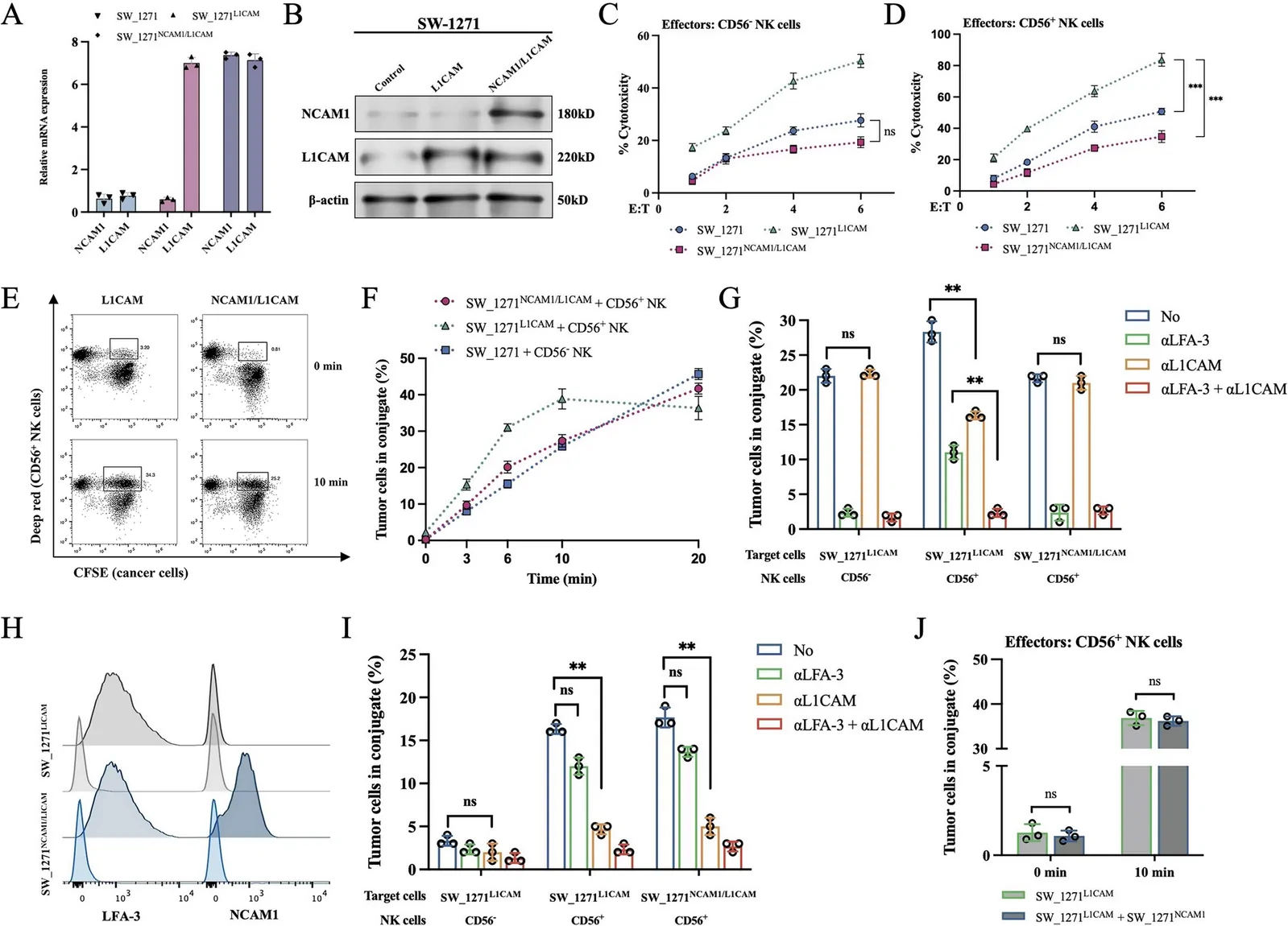
Restriction of conjugate formation by cis-NCAM1/L1CAM interaction. **A**, **B** Expressions of NCAM1 and L1CAM in SW_1271 cell lines, which were stably expressed with NCAM/L1CAM (SW_1271^NCAM/L1CAM^) or L1CAM (SW_1271^L1CAM^), were assessed on transcriptional (**A**) and protein (**B**) levels. **C** The lytic capacity of CD56^−^ (**C**) and CD56^+^ (**D**) NK cells for SW_1271, SW_1271^NCAM/L1CAM^, and SW_1271^L1CAM^ was detected and analyzed, respectively. **E**, **F** CD56^+^ NK cells and tumor cells were previously labeled with fluorescent dyes of different channels, and then their cellular conjugate formations in co-incubation systems were evaluated by flow cytometry at each timepoint. Representative dot plots were shown (**E**) and calculated (**F**). **G** Cellular conjugates formations of NK cells with SW_1271, SW_1271^NCAM/L1CAM^, and SW_1271^L1CAM^ cells was analysed under the treatment with no antibody (No), anti-LFA-3 (αLFA-3), anti-L1CAM (αL1CAM), or a combination of both blocking antibodies (αLFA-3 + αL1CAM). **H** LFA-3 and NCAM1 were silenced in indicated cell lines, followed by flow cytometry validation (**H**). **I** Co-incubation systems were used to analyzed the cellular conjugates of NK cells with SW_1271, SW_1271^NCAM/L1CAM^, and SW_1271^L1CAM^ cells under the treatment with no antibody (No), anti-LFA-3 (αLFA-3), anti-L1CAM (αL1CAM), or a combination of both blocking antibodies (αLFA-3 + αL1CAM). **J** CD56^+^ NK cells were co-incubated with the indicated cell lines, and flow cytometry was then used for detecting conjugate proportions.

Due to CD56 (referred to as “NCAM1” on tumor cells) also being positively expressed on NK cells, we speculated whether the cis-interaction between NCAM1 and its ligand L1CAM on tumor cell prevented the trans-interaction of L1CAM with CD56 on the NK cell surface, thereby reducing the capacity of NK cells to adhere to and lyse NCAM1^+^ tumor cells. We stably expressed L1CAM (SW_1271^L1CAM^) and NCAM1 (SW_1271^NCAM1/L1CAM^) in SW_1271 tumor cells, as assessed by quantitative polymerase chain reaction (qPCR) and western blotting (Fig. 4A, B). To investigate the effect of CD56 on the lytic ability of NK cells, we separated expanded NK cells into CD56^−^ and CD56^+^ cell subpopulations using magnetic beads, followed by co-culturing with the target tumor cells. We found that the lytic capacity of CD56^−^ NK cells was independent of whether tumor cells expressed NCAM1 and L1CAM (Fig. 4C). However, SW_1271^L1CAM^ cells were more prone to be lysed by CD56^+^ NK than those of parental SW_1271 cells (Fig. 4D). While compared to SW_1271^L1CAM^, SW_1271^NCAM1/L1CAM^ cells were resistance to react to CD56^+^ NK cytotoxicity (Fig. 4D), indicating that the expression of NCAM1 on tumor cells attenuated L1CAM-dependent NK cells immunotoxicity. Next, we assessed the proportions of cellular conjugations formed by CD56^+^ NK and tumor cells, both of which were pre-labeled by distinct fluorescence dyes. Flow analyses suggested that SW_1271^NCAM1/L1CAM^ cells formed fewer conjugated cells compared to those of SW_1271^L1CAM^ cells (Fig. 4E, F). Additionally, SW_1271^L1CAM^ cells formed rapid yet less sustained conjugates with CD56^+^ NK cells within 10 minutes (Fig. 4F), which implied that L1CAM seemed to contribute to conjugate formation, while the expression of NCAM1 on the same cell surface could inhibit the formation of conjugates mediated by LICAM between tumor cells and CD56^+^ NK cells.

The binding of CD2 with its ligand LFA-3 was essential for forming cellular adhesion and activating NK cytotoxic function [24]. Adding LFA-3 antibody into the co-incubation system prevented the formation of conjugates between NK cells and tumor cells. Nevertheless, SW_1271^L1CAM^ cells still retained abundant conjugation with CD56^+^ NK when blocking LFA-3, suggesting that L1CAM partially participated in conjugate formation, since combined blockade of LFA-3 and L1CAM could abolish the formation of conjugation (Fig. 4G). Remarkably, blocking of LFA-3 efficiently abolished conjugations formed by SW_1271^NCAM1/L1CAM^ cells and CD56^+^ NK cells, but L1CAM blockade made no difference (Fig. 4G), revealing that NCAM1 expressed by tumor cells prevented L1CAM from contributing to cellular adhesion. To determine whether the L1CAM epitope was masked by cis-binding with NCAM1, we therefore silenced NCAM1 and LFA-3 in both SW_1271^L1CAM^ and SW_1271^NCAM1/L1CAM^ cells in order to release the binding epitope of L1CAM (Fig. 4H). After these processing, adhesion to CD56^+^ NK cells decreased by nearly half in these two cell types, and the whole adhesion proportion obviously decreased after silencing LFA-3 (Fig. 4I). Importantly, the residual conjugates were formed independently by L1CAM, since the conjugate formation were reduced with L1CAM blockade, while no effect for LFA-3 blockade (Fig. 4I). Consistently, SW_1271^L1CAM^ cells that underwent targeted silence could not be adhered by CD56^−^ NK cells anymore (Fig. 4I). These results suggested that L1CAM indeed contributed to the adhesion of tumor cell to NK cells, while the cis-binding of NCAM1 to L1CAM masked the L1CAM epitope, leading to the restriction of L1CAM-mediated tumor cells adhesion to NK cells.

To further confirm whether L1CAM can be masked by NCAM1 expressed on the surface of non-identical tumor cells, we co-cultured CD56^+^ NK cells with SW_1271^L1CAM^ cells as well as an equal mixture of SW_1271^L1CAM^ and SW_1271^NCAM1^ cells. However, the adhesion efficiency of these two groups had no difference (Fig. 4J), demonstrating that the cis-interaction of L1CAM and NCAM1 only occurred on the same tumor cell surface.

### NCAM1 enhances NK cell-mediated cytotoxicity resistance and SCLC stemness by interacting with CHMP4B

**Fig. 5:**
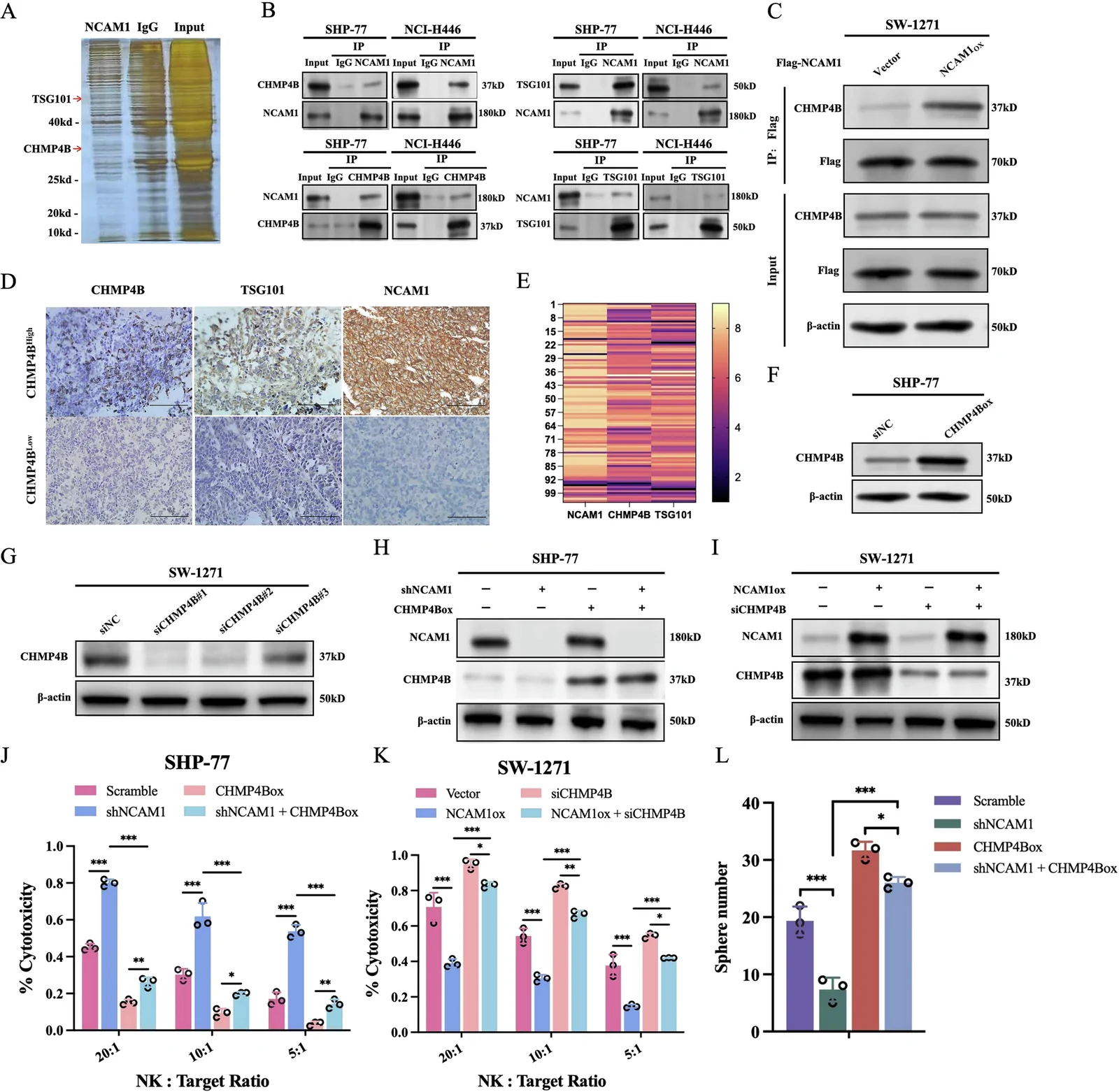
NCAM1 enhances NK cell-mediated cytotoxicity resistance and SCLC stemness by interacting with CHMP4B. **A** NCI-H446 parent cell lysates were immunoprecipitated with an antibody against NCAM1. The arrows suggest proteins interacting with NCAM1 identified by mass spectrometry. **B** NCAM1, CHMP4B, and TSG101 were immunoprecipitated from SHP-77 and NCI-H446 parent cell lysates with antibodies against NCAM1, CHMP4B, and TSG101. Western blotting was then performed, and the interaction of NCAM1 with CHMP4B and TSG101 was confirmed. **C** SW-1271 cells transfected with vector control or NCAM1 overexpression were immunoprecipitated with an antibody against Flag, followed by western blotting to detect the binding of NCAM1 to CHMP4B. A representative image was displayed. **D**, **E** Representative IHC images of CHMP4B, TSG101, and NCAM1 expression (**D**), and the correlations of CHMP4B with TSG101 and NCAM1 expression were illustrated with a heatmap (**B**). Spearman’s rank correlation analysis was used. *n* = 111. **F** Western blotting was performed to assess CHMP4B overexpression in SHP-77 cells. **G** Western blotting was used to determine the silencing efficiencies of CHMP4B in SW-1271 cells. **H** SHP-77 cells were transfected with siNCAM1 or CHMP4B overexpression lentiviruses, followed by Western blotting analysis. **I** SW-1271 cells were transfected with NCAM1 overexpression lentivirus or siCHMP4B plasmid, followed by western blotting analysis. **J**, **K** The lytic capabilities of NK cells for indicated cell lines were assessed and compared at each ETR in SHP-77 cells (**J**) and SW-1271 cells (**K**), respectively. **L** Sphere formation assays were performed in indicated cell lines.

Owing to the cis-binding of NCAM1 with its ligand not preventing all conjugation formation and cytotoxic resistance with NK cells, we therefore hypothesized that there was still an underlying mechanism of resistance to immunotoxicity in the case of adhesion of NCAM1-expressed tumor cells. After co-culture with NK cells, the cell lysate of NCI-H446 was immunoprecipitated with anti-NCAM1 antibody, followed by silver staining of the electrophoretic gel and mass spectrometry identification. Several proteins associated with cytomembrane repair were identified as potential interactors with NCAM1, including the subunits of ESCRT complex such as CHMP4B and TSG101, with high binding abundance (Fig. 5A). Recent research discovered that cancer cells can protect themselves from immune attack via recruiting ESCRT subunits at the immune-synapse structures, which can rapidly repair perforin-induced membrane damage and restricts the entry of cytotoxic granules into cancer cells [25]. As expected, the interactions of NCAM1 with CHMP4B and TSG101 were confirmed in SHP-77 and NCI-H446 cell lines by co-immunoprecipitation (co-IP), irrespective of whether the antibody targeted NCAM1, CHMP4B or TSG101 (Fig. 5B). Additionally, tumor cells overexpressing exogenous NCAM1 exhibited enhanced interaction with CHMP4B compared to those of control cells (Fig. 5C). IHC staining analyses indicated that NCAM1 expression positively correlated with CHMP4B as well as TSG101 in primary SCLC tissues (all *P* value < 0.01) (Fig. 5D, E).

Next, the significance of CHMP4B was investigated in NCAM1-induced cytotoxicity resistance and tumor stemness. The overexpression and silencing efficacy of CHMP4B were detected by western blotting (Fig. 5F, G). CHMP4B was then overexpressed or silenced (using siCHMP4B#1) in SHP-77 cells and SW-1271 cells, which were subsequently co-cultured with NK cells at different ETRs (Fig. 5H, I). CHMP4B-overexpressing cells achieved the maximum resistance to react to immunotoxicity, while overexpressing CHMP4B in NCAM1-silencing cells modestly but significantly restored cytotoxicity compared to that of CHMP4B-overexpressing cells (Fig. 5J). On the contrary, silencing of CHMP4B protected tumor cells from immune attack to the greatest extent, yet simultaneous silencing CHMP4B and overexpressing NCAM1 rendered tumor cells more resistant to NK cell-mediated cytotoxicity (Fig. 5K). Moreover, cells overexpressing CHMP4B could form more tumor spheres, and CHMP4B overexpression combined with NCAM1 silencing reduced sphere formation of tumor cells compared with cells overexpressing CHMP4B (Fig. 5L). These results demonstrated that ESCRT had crucial effects on immune cytotoxicity resistance and tumor stemness, and the interaction of NCAM1 and ESCRT subunits may recruit more CHMP4B and TSG101 proteins to repair perforin-triggered membrane damage, thereby leading to additional toxicity resistance of tumor cells to NK cells.

### Tumors with NCAM1^high^CHMP4B^high^ expression predict poor survival of SCLC

**Fig. 6:**
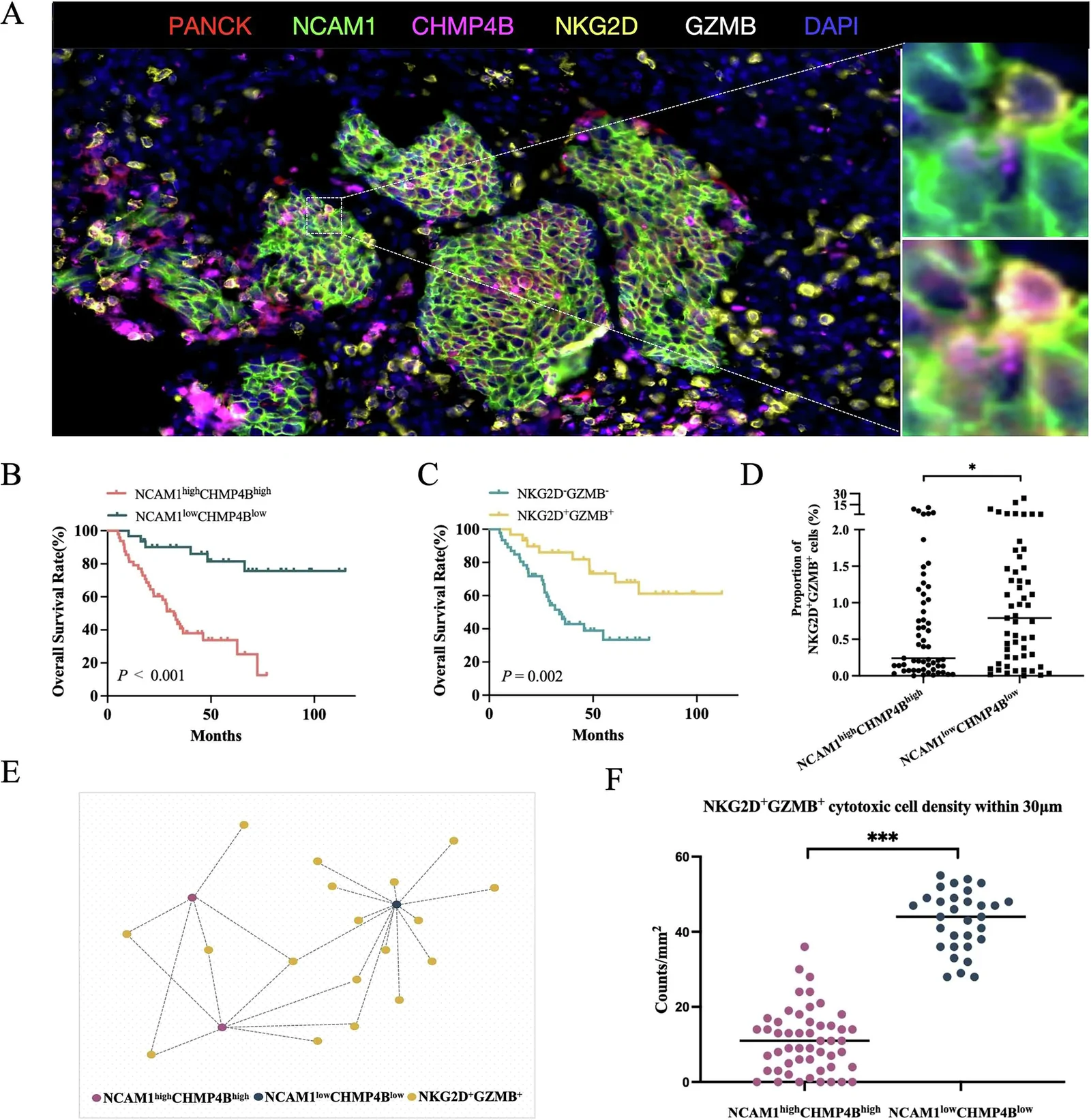
Tumors with NCAM1^high^CHMP4B^high^ predict poor survival of SCLC. **A** Multiplex fluorescent IHC staining of PANCK, NCAM1, CHMP4B, NKG2D, and GZMB. Magnified images showed the conjugation of NKG2D^+^GZMB^+^ NK cells to NCAM1^+^ tumor cells, and CHMP4B were enriched in cellular junctions or immune-synapse structures and was co-localized with NCAM1. **B** Kaplan–Meier analysis for OS was performed in NCAM1^high^CHMP4B^high^ and NCAM1^low^CHMP4B^low^ subsets by the Log-Rank test. **C** Kaplan–Meier analysis for OS was performed in NKG2D^−^GZMB^−^ and NKG2D^+^GZMB^+^ subsets by the log-rank test. **D** Proportion of NKG2D^+^GZMB^+^ cytotoxic cells was analyzed and compared in NCAM1^high^CHMP4B^high^ and NCAM1^low^CHMP4B^low^ subsets. Student’s *t* test was used. **E** Schematic illustration for the distribution of NKG2D^+^GZMB^+^ cytotoxic cells around NCAM1^high^CHMP4B^high^ and NCAM1^low^CHMP4B^low^ tumor cells. **F** Density of NKG2D^+^GZMB^+^ cytotoxic cells within 30 µm from NCAM1^high^CHMP4B^high^ and NCAM1^low^CHMP4B^low^ tumor cells was analyzed and compared by student’s *t* test.

The activation of NKG2D and the release of GZMB are of crucial importance in the NK cell-mediated tumor lysis process. To examine the associations of NCAM1 and CHMP4B expression with immune cytotoxicity resistance, we performed multiplex IHC staining using constructed tumor microarrays. NKG2D^+^GZMB^+^ cells were observed infiltrating SCLC tumor specimens (Fig. 6A). NCAM1-expressed tumor cells were adhered by NKG2D^+^GZMB^+^ cytotoxic immune cells, and CHMP4B exhibited fluorescent co-localization with tumorous NCAM1 at the immune-synapse structures (Fig. 6A), which demonstrated that the recruitment of CHMP4B may essentially contribute to resisting immunotoxicity. Analyses of these assays further manifested that patients with NCAM1^high^CHMP4B^high^ expression had a significantly shortened OS compared to patients with NCAM1^low^CHMP4B^low^ subgroup (Fig. 6B). Conversely, the infiltration of NKG2D^+^GZMB^+^ cells was beneficial to prolong patients’ OS than those with NKG2D^−^GZMB^−^ cells infiltration (Fig. 6C). Moreover, NKG2D^+^GZMB^+^ cytotoxic immune cells seemed to preferentially infiltrate into specimens with NCAM1^low^CHMP4B^low^ expression (Fig. 6D). The infiltration density of NKG2D^+^GZMB^+^ cells within 30 µm of tumor cell with NCAM1^high^CHMP4B^high^ expression was also reduced compared to tumor cells with NCAM1^low^CHMP4B^low^ expression (Fig. 6E, F). These results highlighted that the concurrent high expression of CHMP4B and NCAM1 had an adverse role in patients’ survival as well as the infiltration of cytotoxic immune cells.

## Discussion

The clinical application of immunotherapy has dramatically transformed treatment strategy for more than 20 cancer types in the last decade, while only a minority of patients with SCLC achieve durable response and survival benefit [4]. Although restoring the functions of immune cells contributes to tumor elimination, there residues tumor cells acquiring resistance to immune responses via complex interactions with immune cells, leading to rapid tumor progression [26]. Cancer stem cells have similar properties to normal stem cells and are capable of evading antitumor immunity through multiple intrinsic and extrinsic mechanisms, including SCLC [27]. Therefore, it is imperative to provide optimal therapeutic targets to overcome stemness properties and immune resistance for SCLC. In this study, we have demonstrated that aberrant expression of NCAM1 not only prevents the adhesion of cancer cells to NK cells via cis-binding to its ligand L1CAM, but also protects cancer cells from immune attack by interacting with ESCRT subunits TSG101 and CHMP4B even if cancer cells were adhered by NK cells, eventually resulting in immune cytotoxicity resistance and stemness enhancement of SCLC. The graphical abstract illustrates the overall workflow of this study.

Aberrant NCAM1 expression was capable of triggering intracellular signaling by cis- or trans-interacting with its various ligands, thereby promoting cancer invasion and metastasis [13]. SCLC originated from pulmonary neuroendocrine cells, and the positive expression of NCAM1 was regularly used for histologic diagnosis of SCLC. SCLC cells with a high degree of stemness concurrently expressed NCAM1, and NCAM1-positive SCLC cells had higher capability of clonal formation [28]. Moreover, the positive expression of NCAM1 on tumor cells could prevent conjugate formation with NK cells and reduce susceptibility to NK cell lysis [29]. These findings indicated the role of NCAM1 in contributing tumor stemness and blocking conjugate formation and immune attack. Further investigation of potential mechanisms of these phenotypes was conducive to expanding our knowledge of NCAM1 in cancer progression of SCLC.

Based on mass spectrometry identification, we recognized several proteins involved in plasma membrane repair, including ESCRT subunits, which may interact with NCAM1. The rapid repair of cell membrane damage induced by cytotoxic chemoradiotherapy and immunotherapy conferred cancer cells more prone to survive, thereby resulting in tumor progression [30, 31]. More efficient capability of plasma membrane repair could be activated by cancer stem cells, contributing to their intrinsic self-renewing ability [32, 33]. CHMP4B and TSG101, members of ESCRT, can be recruited to the binding sites of cytotoxic T lymphocytes in cancer cells, thereby restricting the entry of granzymes into the cytoplasm and may enable cancer cells to resist cytolytic attack [25]. Extracellular vesicles derived from cancer stem cells could enrich more phosphorylated PKM2 via TSG101 to transmit stemness traits and thus replenish the stem cell pool in non-SCLC [34]. Besides, TSG101 was also required for the assembly of LC3C with its upstream regulators, which constituted a human-specific autophagic program relevant to cancer stem cells [35]. The decreased assembly capability of the ESCRT complex via CHMP4B attenuated stemness and progression of hepatocellular carcinoma [36]. Consistently, our data demonstrated that CHMP4B overexpression increased tumor stemness and reduced NK cell-mediated toxicity, while silencing NCAM1 weakened CHMP4B-dependent stem-like feature enhancement and cytotoxic resistance, implying that targeting ESCRT subunit CHMP4B was promising for overcoming cancer stemness and treatment resistance. Given that tumor stem cells were more resistant to immune cytotoxicity, we further investigated whether the role of NCAM1 in NK cell-mediated cytotoxicity resistance was associated with its contributing role in self-renewal. We found that SOX2-overexpressing cells achieved the maximum resistance to react to immunotoxicity. Compared with SOX2-overexpressing cells, tumor cells with SOX2 overexpression in the NCAM1-silencing cells showed enhanced resistance to immune cytotoxicity, with significantly lower cytotoxicity compared to NCAM1-silencing cells (Supplementary Fig. 1). These results suggested that tumor-promoting NCAM1 could not only facilitate cell self-renewal, but also attenuate NK cell-mediated cytotoxicity, with different contributing degrees.

Apart from the directly lytic capability of NK cells, related chemokine secretion and target cell adhesion also crucially influenced host antitumor immunity. More secretion of chemokines CXCL9/10 enhanced NK cell infiltration, which was associated with prolonged survival in patients with pancreatic ductal adenocarcinomas [37]. An improved migratory ability of ex-vivo expanded NK cells towards cancer cells was dependent on tumor-derived CXCL10, and NK cells transfer significantly reduced tumor burden and increased survival of CXCL10-transfected melanoma tumors [38]. Through patients’ specimens and in vitro experiments, we confirmed the unfavorable roles of NCAM1 in cytotoxic immune cells infiltration, CXCL9/10 secretion, granzyme release, and clinical outcomes, which provided synergetic mechanisms in NCAM1-induced immune cytotoxicity resistance. Cancer cells adhered by cytotoxic immune cells were prerequisites for the formation of tight cellular junctions or immune-synapse structures and the following attack by cytotoxic granzymes. Compared to healthy individuals, NK cells with adhesion defects were accumulated in patients with multiple myeloma and were associated with a shorter OS [39]. The cis-PD-L1/CD80 interactions on the same cell surface of primary activated dendritic cells blocked PD-L1 from binding with its ligand PD-1 on T cells, thereby maintaining T cell functions in antitumor immunity [40, 41]. As a ligand for NCAM1, the expression of L1CAM is linked to tumor invasiveness, chemotherapy resistance, and poor survival in many cancers [42, 43]. Binding of NCAM1 to its ligand L1CAM in cis or trans on neuron cells has been previously well-established in promoting cellular adhesion and tight junctions between neurons [44]. Given that CD56 (NCAM1) is also expressed on NK cells, we therefore speculated that cis-NCAM1/L1CAM interaction may block tumor cells from adhering to NK cells and thereby leading lytic resistance. Succeeding functional data demonstrated the cis-interaction of NCAM1 with L1CAM on the same cell surface of tumor cells, and L1CAM was preferentially bound by NCAM1 when CD56 was concurrently expressed by NK cells. This work further supplemented a potential mechanism of tumorous NCAM1 as a negative regulator of NK cell function.

Our findings provided a profound understanding of the functions of NCAM1 in enhancing tumor stemness and resisting NK cell cytolysis in SCLC and offer a potential target for improving treatment efficacy. Meanwhile, several limitations of this research should be mentioned herein. Firstly, we only used NK cells to perform this study, and the role of NCAM1 in the lytic resistance to cytotoxic T cells and chemoradiotherapy was not evaluated. It has been reported that the tumorigenesis and drug resistance in acute myeloid leukemia were involved in the aberrant expression of NCAM1 [45]. Further investigations were necessary to explore the effects of NCAM1 on chemoradiotherapy and cytotoxic T cell function in SCLC and reconfirm the potential resistance mechanisms. Second, the cis-binding of NCAM1 with its heterophilic ligand L1CAM was only considered in this study, and we did not investigate whether NCAM1 had a homophilic interaction with itself in cis [13]. Moreover, a specific inhibitor targeting CHMP4B needed to be developed and evaluated to maximally overcome cancer stemness and enhance treatment efficacy in translational research and clinical trials.

## Conclusions

We demonstrated the adverse effect of NCAM1 on SCLC progression, stemness, and lytic sensitivity to NK cells. Mechanistically, NCAM1 not only bound to its ligand L1CAM in cis to prevent tumor cells from being adhered by NK cells, but it also protects tumor cells from immune attack and stem cell renewal by interacting with ESCRT members, which were further validated in clinical specimens. These findings shed light on novel molecular mechanisms of NCAM1 in SCLC progression and provide a novel target of CHMP4B against stemness and immunoresistance in the field of SCLC.

## Materials and methods

### Cell culture and lentivirus infection

Human SCLC cell lines SHP-77, NCI-H446 and SW-1271 were purchased from American Type Culture Collection (Manassas, VA, USA) and confirmed mycoplasma-negative prior to experiments. These cells were cultured in the recommended medium of RPMI-1640 or IscoveL-15 supplemented with 10% fetal bovine serum and 1% penicillin/streptomycin. The validated short-hairpin DNA (shRNAs) of NCAM1 as well as overexpression plasmids of NCAM1, CHMP4B and L1CAM were purchased from Genechem (Shanghai, China). Small interfering RNAs (siRNAs) of CHMP4B were obtained from RiboBio (Guangzhou, Guangdong, China). The mix of lentiviral plasmid and packaging plasmids was co-transfected into 293 T for recombinant lentivirus generation, which was used to transfect targeted cells combined with infection enhancer following the manufacturer’s instructions. The transfection of empty scramble/vector into targeted cells was applied as the control. Three siRNA sequences for targeting CHMP4B were as follows: siCHMP4B#1 (5′-GACGGCACATTATCAACCA-3′), siCHMP4B#2 (5′-GGACATCGATAAAGTTGAT-3′), and siCHMP4B#3 (5′-GGCGGAATTAGAAGAACTA-3′), and the most efficient one was employed to perform relevant assays. The siRNA sequence for targeting LFA-3 was 5′-CAACUU AACAUCAUCAGAU-3′.

### Flow cytometry analysis

Tumor single-cell suspensions were prepared from tumor tissues by enzymatic digestion. Cells were washed and resuspended in FACS buffer. For surface staining, 1–10 × 10^6^ cells were incubated with corresponding antibodies. For intracellular cytokine staining, cells were stimulated with PMA/ionomycin and brefeldin A for 4 hours, then fixed, permeabilized, and stained with corresponding antibodies. These following antibodies were used at a concentration of 2.5 μL per 100 μL cell suspension, including: NCAM1-PE/Cyanine7 (Cat#364510, Biolegend), SOX2-PE (Cat#656103, Biolegend), CD24-FITC (Cat#311104, Biolegend), CD44-APC (Cat#397505, Biolegend), CD3-FITC (Cat#317306, Biolegend), NKG2D-APC/Cyanine7 (Cat#320823, Biolegend), Perforin-PE (Cat#308105, Biolegend), GZMB-PE (Cat#372207, Biolegend), LFA-3-APC (Cat#330917, Biolegend). All samples were detected by flow cytometry using FlowJo V.10.0. Representative gating strategies are shown in Supplementary Fig. 2.

### Western blotting

Cells were harvested and lysed with a mix of radioimmunoprecipitation assay (RIPA) lysis buffer (P0013B, Beyotime, Shanghai, China) and protease inhibitor phenylmethylsulfonyl fluoride (PMSF, ST507, Beyotime, Shanghai, China), followed by sonication. After further lysis on ice for 30 min, samples were centrifuged at 12,000 × *g* for 15 min at 4 °C. The supernatants were then collected to assess protein concentration using Pierce™ BCA Protein Assay Kit (23225, Thermo Fisher Scientific, Waltham, MA, USA). Sodium dodecylsulfate (SDS)-polyacrylamide gel electrophoresis (PAGE) loading buffer was used to sufficiently denature samples after heating at 95 °C for 5 min. Aliquots of samples were subjected to gel electrophoresis using Gel Kit (PG112, EpiZyme, Shanghai, China) and were then transferred onto polyvinylidene fluoride (PVDF) membrane (IPVH00010, Millipore, Merck, Germany). Ultra-sensitivity chemiluminescence (ECL) buffer (AP34L024, Life-iLab, Shanghai, China) was applied for optical detection (The full-length uncropped original western blots are shown in Original data). The following antibodies were used: NCAM1 (99746S, Cell Signaling Technology), ABCG2 (ab207732, Abcam), SOX2 (ab92494, Abcam), CD133 (ab216323, Abcam), L1CAM (ab270455, Abcam), Flag (ab205606, Abcam), CHMP4B (ab135154, Abcam), TSG101 (ab125011, Abcam), β-actin (4970 T, Cell Signaling Technology), Anti-mouse IgG, HRP-linked Antibody (7076P2, Cell Signaling Technology), Anti-rabbit IgG, HRP-linked Antibody (7074P2, Cell Signaling Technology).

### Tumorsphere assay

Ultra-low attachment cell culture plate coating solution (C0365, Beyotime, Shanghai, China) was applied for pre-coating 6-well plates, which were used for seeding tumor cells in serum-free tumorsphere medium (P2401, QiDa Biotechnology, Shanghai, China). The number of tumor spheres with a diameter ≥70μm were counted under optical microscope after 10 days.

### Cell apoptosis assay

NK cells were collected and stained with NKG2D-APC/Cyanine7, followed by co-cultured with tumor cells at a ratio of 1:2 for 1 h. Then, tumor cells were harvested for apoptosis assay using Annexin V-FITC Apoptosis Detection Kit (C1062M, Beyotime, Shanghai, China) following the manufacturer’s instructions. All samples were detected by flow cytometry using FlowJo V.10.0. NKG2D^+^ cells were gated out of the NK cells for the final analysis. NKG2D was used to gate out the NK cells, aiming to eliminate confounding by the presence of effector cells.

### NK cells isolation and ex-vivo activation and expansion

Human peripheral blood mononuclear cells from healthy donors (*n* = 10) were obtained from Tianjin Blood Center, with an equal sex distribution and ages ranging from 18 to 36 years. After isolating by lymphocyte separation medium (abs930, Absin, Shanghai, China), samples were then magnetically sorted to acquire NK cells using NK Cell Isolation Kit (130-092-657, Miltenyi Biotec, Germany). Natural Killer Cells Induction Culture Kit 2.0 (6813533, Dakewe, Shenzhen, China) were applied for ex vivo activation and expansion according to the manufacturer’s instructions. All experiments were repeated with NK cells from independent donors, and all data were averaged per donor.

### Lactate dehydrogenase detection

Tumor cells were harvested and seeded in a 96-well plate with a round bottom, with 10,000 cells/well. NK cells were then mixed with tumor cells according to distinct ETRs to a final volume of 200 μl. After centrifugation at 1000 rpm for 5 min, the plate was cultured at 37 °C for 3.5 hours. CytoTox 96® Non-Radioactive Cytotoxicity Assay (G1780, Promega, USA) was applied for lactate dehydrogenase (LDH) detection following the recommended instructions.

### Realtime cellular analysis

Tumor cells were collected and passaged in RTCA E-Plate Inserts (300600890, Agilent, California, USA) with 200,000 cells/well. At 24 hours after the cells adhered, NK cells were then added to the plate at an ETR of 5:1, followed by gentle mixing. The proliferation curves of tumor cells were dynamically detected by realtime cellular analysis using RTCA Software.

### Live cell imaging

Approximately 1 × 10^5^ tumor cells were seeded in a dish with a diameter of 3 cm and cultured at 37 °C for 24 h. CFSE (65-0850-84, Thermo Fisher Scientific, Waltham, MA, USA) was used to label tumor cells at room temperature for 10 min. After terminating staining using complete culture medium at 4 °C for 20 min, a tenfold quantity of NK cells was added to the culture dish, followed by live cell imaging for 24 hours.

### SEM imaging

Tumor cells were harvested and seeded in Poly-L-lysine-coated coverslips in 24-well plates (WHB-24-CS-LC, WHB Scientific, Shanghai, China). After cells adhered the following day, an equal quantity of NK cells was transferred onto plates at 37 °C for 1 hour, followed by fixation using 2.5% glutaraldehyde (P1126, Solarbio Life Science, Beijing, China) at room temperature for 2 hours and then 4 °C for 2 hours. After adequate washing with phosphate buffer at pH 7.4, samples were fixed with 1% osmium tetroxide at room temperature for 2 hours. Sequential dehydration was performed for 15 minutes each with ethanol at gradient concentrations (30%, 50%, 70%, 80%, 90%, 95%, 100%, 100%) and isoamylacetate. Then the samples were dried in a critical point dryer (K850, Quorum) and sputtered with gold-palladium coating for 30 seconds (MC1000, HITACHI), followed by SEM imaging (SU8100, HITACHI).

### Chemokines secretion assay

Aliquots of NK cells and tumor cells were incubated in a 96-well plate for 24 hours, and then the supernatants were collected, and the levels of CXCL9 and CXCL10 were detected using Human CXCL9 ELISA Kit (ab100595, Abcam) and Human CXCL10 ELISA Kit (ab83700, Abcam).

### Quantitative realtime PCR

Total RNA was extracted from tumor cells using Trizol reagent (R0016, Beyotime, Shanghai, China) according to the manufacturer’s instructions, followed by concentration detection. BeyoRT™ III First Strand cDNA Synthesis Kit (D7178, Beyotime, Shanghai, China) was used to obtain cDNA from 1 µg of RNA per sample, which was then amplified using BeyoFast™ AfterSYBR Green qPCR Mix (D7260, Beyotime, Shanghai, China). Relative expressions were assessed using the Δ Ct method with the normalization of gene coding glyceraldehyde-3-phosphate dehydrogenase (GAPDH) for human. The following primer sequences were used: NCAM1 (5’-CATCACCTGGAGGACTTCTACC-3’ and 5’-CCAAGGACTCCTGCCCAATG-3’), L1CAM (5’-ACGAGGGATGGTGTCCACTTCAAA-3’ and 5’-TTATTGCTGGCAAAGCAGCGGTAG-3’), GAPDH (5’-GGTATGACAACGAATTTGGC-3’ and 5’-GAGCACAGGGTACTTTATTG-3’).

### Cellular conjugate formation

Tumor cells were harvested and stained with CFSE, and a fivefold quantity of NK cells was collected for staining with CellTracker™ Deep Red (A66433, Thermo Fisher Scientific, Waltham, MA, USA). These two types of cells were then co-cultured to form conjugates for 3 min, 6 min, 10 min, and 20 min, respectively. Flow cytometry was used for detecting conjugates formed at each time point.

### Co-IP assay

After co-cultured with NK cells for 1 hour, tumor cells were harvested and lysed by a mix of cell lysis buffer (P0013, Beyotime, Shanghai, China) and PMSF on ice for 30 min. After centrifugation at 12,000 × *g* for 15 min, the cell supernatant was acquired. Protein A/G magnetic beads (HY-K0202, MedChemExpress) were washed three times with cell lysis buffer. Then the beads were incubated with the antibody on a flip mixer at room temperature for 30 min, and anti-IgG antibody was used as a control. The antibody-conjugated magnetic beads were washed three times with cell lysis buffer, and then the prepared protein supernatant was added at 4 °C for 2 hours on a flip mixer. After washing three times using cell lysis buffer, SDS-PAGE protein loading buffer was added to denature at 95 °C for 5 min, followed by western blotting detection.

### Electrophoretic gel silver staining

After electrophoresis, the protein gel was fixed overnight in solutions containing 50% ethanol and 10% acetic acid, followed by sequential washing with 30% ethanol and Milli-Q grade pure water. Silver staining of protein gel was then performed using the Fast Silver Stain Kit (P0017S, Beyotime, Shanghai, China) according to the manufacturer’s instructions.

### Mass spectrometry analysis

Liquid chromatography tandem mass spectrometry experiments were performed on a Q Exactive HF-X mass spectrometer (Thermo Fisher Scientific) equipped with an Ultimate 3000 nano-liquid chromatography system (Thermo Fisher Scientific). Peptides were trapped on a homemade reversed-phase C18 precolumn (100 μm i.d. × 2 cm, 3 μm particle size, 120 A pore size) and separated on an analytical column (150 μm i.d. × 30 cm, 1.9 μm particle size, 120 A pore size) using a binary gradient of 0.1% formic acid in water (solvent A) and 80% acetonitrile with 0.1% formic acid (solvent B) delivered at 600 nL/min. The linear gradient was programmed as follows: 6% B (0–10 min), 6–10% B (10–15 min), 10–30% B (15–80 min), 30–40% B (80–90 min), 40–95% B (90–90.1 min), held at 95% B (90.1–95 min), and returned to 6% B (95–100 min). Full MS scans were acquired in the Orbitrap at a resolution of 120,000 (at m/z 200) over a mass range of m/z 340–1100 with an AGC target of 5 × 10^5^, maximum injection time of 80 ms, and charge state inclusion of 2–6. Precursor ions were fragmented by higher-energy collisional dissociation at a normalized collision energy of 27% using data-independent acquisition mode with 60 sequential isolation windows of 12.5 m/z each, and MS/MS spectra were recorded in the Orbitrap at a resolution of 30,000 with an AGC target of 3 × 10^6^ and maximum injection time of 45 ms. Dynamic exclusion was set to 18 s with a mass tolerance of ±7 ppm.

### Immunohistochemistry and multiplex fluorescent immunohistochemistry

Consecutive tumor sections of 138 SCLC cases were used for staining in the following experiments. After deparaffinization and antigen retrieval, tumor sections were incubated with corresponding antibodies at 4 °C overnight. Appropriate secondary antibody was added onto sections at room temperature for 1 hour the following day, and 3,3′-diaminobenzidine was used for coloration. Staining results were calculated under an optical microscope by the product of staining intensity (0: negative, 1: weak, 2: moderate, 3: strong) and positive proportion. The median was chosen as the cutoff value, and specimens were therefore dichotomized to tumors with high expression (≥median) or low expression (<median). Multiplex fluorescent immunohistochemistry (mIHC) was performed using Absin 6-Color IHC Kit (abs50014, Absin Biotechnology, China) following the manufacturer’s protocol. The antibodies used in mIHC staining were as followed: PANCK (abs123684, Absin, 1:400), NCAM1 (abs171540, Absin, 1:200), CHMP4B (13683-1, Proteintech, 1:200), NKG2D (ab302907, Abcam, 1:2000), and GZMB (abs171433, Absin, 1:200). Spectral unmixing was achieved by applying a single dye at a high concentration and scanning the corresponding channel and adjacent channels, aiming to detect crosstalk issues and ensure that the emitted light of the dye did not cause crosstalk to adjacent channels under the conditions of the scanner filter. Pannoramic MIDI II (3DHISTECH) and Indica Halo software were applied for fluorescent whole-slide scanning and quantitative image analysis for pathological sections.

### Statistical analysis

Statistical analyses were conducted using GraphPad Prism v10.0 (GraphPad Software Inc., San Diego, CA, USA) and SPSS v26.0 (IBM Corporation, Armonk, NY, USA). The data from the in vitro experiment were performed at least three times independently and were presented as mean ± standard deviation (SD). Normality was assessed using the Shapiro–Wilk test, and the data were found to be normally distributed (*P* > 0.05). Prior to parametric testing, homogeneity of variance was assessed using Levene’s test. All compared groups exhibited similar variance (*P* > 0.05), satisfying the assumption for two-tailed Student’s *t* test and one-way analysis of variance. In cases where variances were unequal, Welch’s *t* test was applied with Dunnett’s T3 post hoc correction. The correlations of continuous variables were assessed by Pearson Correlation Coefficient. Survival curves were delineated with the Kaplan–Meier method and compared with the Log-Rank test. *P* values below 0.05 of the two-sided test were considered statistically significant, and significance levels were denoted as follows: *P < 0.05; **P < 0.01; ***P < 0.001; ns, non-significant.

## Supplementary information

Supplementary Table

Supplementary Figure

Original Data

## Data Availability

The data supporting the conclusions of this article will be made available by the authors on reasonable request.
