# Supplementary material for: NCAM1 potentiates small cell lung cancer stemness and resistance to NK cell-mediated cytotoxicity
Source: Cell Death Dis. 2026 Aug 15;17(1):960. doi: 10.1038/s41419-026-09182-2 (PMC13754799; doi:10.1038/s41419-026-09182-2)
Supplement: Supplementary file 1 [file 41419_2026_9182_MOESM1_ESM.docx]

**Supplementary Table**

**Table 1:** The clinicopathological charateristics of small cell lung cancer patients

| Characteristics |  | SCLC (N%) |
| --- | --- | --- |
| Age, years |  |  |
| ≥65 |  | 37 (26.8) |
| <65 |  | 101 (73.2) |
| Gender |  |  |
| Female |  | 31 (22.5) |
| Male |  | 107 (77.5) |
| Smoking history |  |  |
| Smoker |  | 114 (82.6) |
| None-smoker |  | 24 (17.4) |
| T stage |  |  |
| T1-T2 |  | 115 (83.3) |
| T3-T4 |  | 23 (16.7) |
| N stage |  |  |
| N0-N1 |  | 94 (68.1) |
| N2-N3 |  | 44 (31.9) |
| Pathological stage |  |  |
| Ⅰ-Ⅱ |  | 87 (63.0) |
| Ⅲ-Ⅳ |  | 51 (37.0) |
| Adjuvant chemotherapy |  |  |
| Yes |  | 85 (61.6%) |
| No |  | 53 (38.4) |

Abbreviations: SCLC = small cell lung cancer.
