# Supplementary material for: NCAM1 potentiates small cell lung cancer stemness and resistance to NK cell-mediated cytotoxicity
Source: Cell Death Dis. 2026 Aug 15;17(1):960. doi: 10.1038/s41419-026-09182-2 (PMC13754799; doi:10.1038/s41419-026-09182-2)
Supplement: Supplementary file 2 [file 41419_2026_9182_MOESM2_ESM.docx]

**Supplementary Figure**

Fig 1: The lytic capabilities of NK cells for indicated cell lines were assessed and compared at each ETRs in SHP-77 cells.

Fig 2: A Representative gating strategy.
