# Supplementary material for: NCAM1 potentiates small cell lung cancer stemness and resistance to NK cell-mediated cytotoxicity
Source: Cell Death Dis. 2026 Aug 15;17(1):960. doi: 10.1038/s41419-026-09182-2 (PMC13754799; doi:10.1038/s41419-026-09182-2)
Supplement: Supplementary file 3 [file 41419_2026_9182_MOESM3_ESM.docx]

Original blots:
